# Retinal Patterns and the Role of Autofluorescence in Choroideremia

**DOI:** 10.3390/genes15111471

**Published:** 2024-11-14

**Authors:** Federica E. Poli, Robert E. MacLaren, Jasmina Cehajic-Kapetanovic

**Affiliations:** 1 Nuffield Laboratory of Ophthalmology, University of Oxford, Oxford OX3 9DU, UK; 2 Oxford Eye Hospital, Oxford University Hospitals NHS Foundation Trust, Oxford OX3 9DU, UK; 3 Royal Berkshire NHS Foundation Trust, Reading RG1 5AN, UK

**Keywords:** choroideremia, autofluorescence, gene therapy

## Abstract

Background: Choroideremia is a monogenic inherited retinal dystrophy that manifests in males with night blindness, progressive loss of peripheral vision, and ultimately profound sight loss, commonly by middle age. It is caused by genetic defects of the *CHM* gene, which result in a deficiency in Rab-escort protein-1, a key element for intracellular trafficking of vesicles, including those carrying melanin. As choroideremia primarily affects the retinal pigment epithelium, fundus autofluorescence, which focuses on the fluorescent properties of pigments within the retina, is an established imaging modality used for the assessment and monitoring of affected patients. Methods and Results: In this manuscript, we demonstrate the use of both short-wavelength blue and near-infrared autofluorescence and how these imaging modalities reveal distinct disease patterns in choroideremia. In addition, we show how these structural measurements relate to retinal functional measures, namely microperimetry, and discuss the potential role of these retinal imaging modalities in clinical practice and research studies. Moreover, we discuss the mechanisms underlying retinal autofluorescence patterns by imaging with a particular focus on melanin pigment. Conclusions: This could be of particular significance given the current progress in therapeutic options, including gene replacement therapy.

## 1. Introduction

Choroideremia (OMIM 303100) is an X-linked recessive inherited retinal dystrophy that leads to progressive and severe sight loss, usually by middle age. The most common underlying aetiology is a loss of function mutation in the *CHM* gene, which is located on chromosome *Xq21.2*. This causes a deficiency in Rab-escort protein-1 (REP1) [1,2,3], which plays an essential role in the regulation of intracellular vesicular trafficking and prenylation of Rab GTPases [4]. Retinal degeneration in choroideremia primarily starts in the retinal pigment epithelium (RPE). This is followed by degeneration of the choroid and photoreceptor death, which occurs predominantly as a consequence of RPE loss, although a degree of independent photoreceptor loss may be taking place [5].

**Figure 1:**
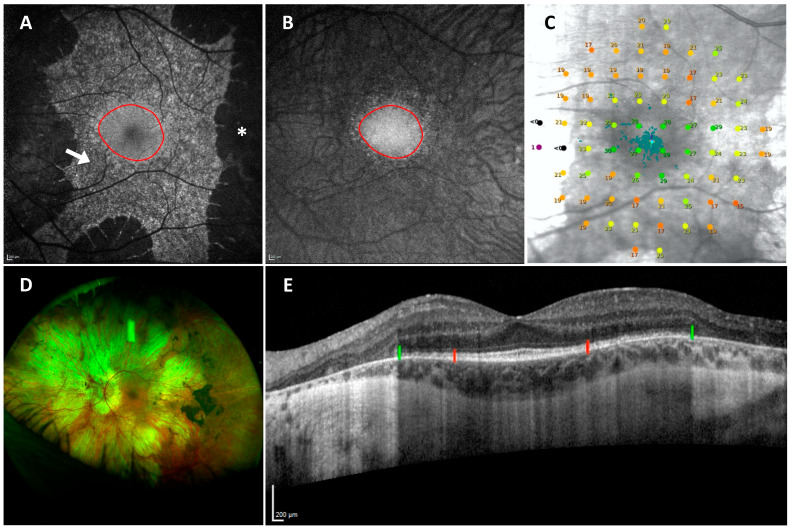
Multimodal retinal imaging and retinal sensitivity testing of the left eye in a patient with choroideremia. Panel (**A**): short-wavelength blue autofluorescence (BAF) imaging demonstrating a smooth zone centrally around the fovea (outlined in red), a mottled zone eccentric to this (arrow), and an atrophic area peripherally (asterisk). Panel (**B**): Near-infrared autofluorescence (NIR-AF) imaging. The red outline demonstrates the correspondence between the smooth zone on BAF and the area of homogenous NIR-AF signal. Panel (**C**): microperimetry map taken with the MAIA microperimeter (CenterVue, Padova, Italy). This shows better function closer to the fovea (green points), impaired but present function in the area corresponding to mottled BAF (yellow-orange points), and no function outside the island of BAF (black points). Panel (**D**): Colour fundus photograph. Panel (**E**): trans-foveal optical computed tomography (OCT) image showing preserved ellipsoid zone within the area of smooth BAF/homogenous NIR-AF (between the two red markers), disrupted ellipsoid zone in the area of mottled BAF/absent NIR-AF (between the red and green markers), and absent ellipsoid zone with retinal and choroidal degeneration in the peripheries (outside the green markers). BAF, NIR-AF and OCT images were taken with Heidelberg Spectralis, Heidelberg Engineering GmbH, Heidelberg, Germany.

**Figure 2:**
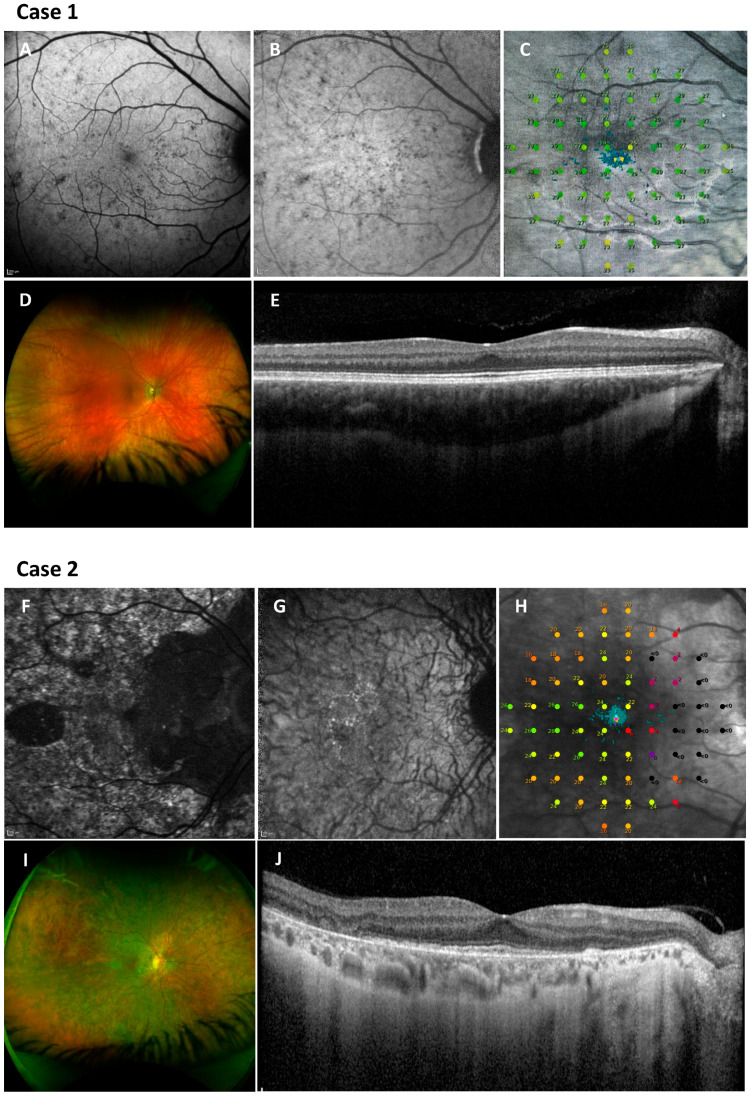
Multimodal retinal imaging and retinal sensitivity testing in two female carriers. Case 1: 20-year-old asymptomatic female carrier, VA 6/5 (colour fundus photograph, (**D**)). Case 2: 44-year-old affected female carrier with male pattern choroideremia, VA 6/9 (colour fundus photograph, (**I**)). Fundus autofluorescence imaging demonstrates early ‘salt and pepper’ mottled appearance (BAF) (**A**) and normal smooth zone (NIR-AF) (**B**) in Case 1, compared with more advanced mottling and areas of atrophy (BAF) (**F**) and near-absent autofluorescence smooth zone (NIR-AF) (**G**) in Case 2. Microperimetry maps (MAIA microperimeter, CenterVue, Padova, Italy) show near normal sensitivity in the asymptomatic carrier (**C**) but reduced sensitivity over the mottled zone with absent sensitivity over the atrophic areas in the affected carrier typical of male pattern choroideremia (**H**). OCT imaging shows an intact ellipsoid zone in Case 1 (**E**), whilst disruption of the ellipsoid zone is visible in Case 2 (**J**).

Clinically, choroideremia manifests in males commonly with nyctalopia from childhood, which often goes unnoticed for years, indicative of rod photoreceptor dysfunction. Visual field restriction follows, and ultimately, visual acuity deteriorates. The pattern of degeneration is characteristically centripetal, starting from the peripheries and spreading centrally, often leaving a preserved central retinal island until the later stages [6]. On fundoscopic examination, peripheral pigment accumulation is seen in the early stages. As the disease progresses, degeneration of the RPE and choriocapillaris expose large choroidal vessels, and in end-stage disease, the sclera becomes visible due to complete RPE and choroid atrophy [7] (Figure 1). Good visual acuity is usually retained until the fourth to fifth decade of life, when the disease progression encroaches on the fovea, causing a sharp decline in visual acuity [6,8]. This supports the hypothesis that cone photoreceptors are not as severely affected as rod photoreceptors by the REP1 deficiency. However, retinal changes are detectable from the early stages of the disease, when retinal thickening occurs, possibly secondary to Müller cell activation and hypertrophy in response to photoreceptor stress. Loss of RPE pigment and photoreceptor death is then followed by retinal disorganization and remodelling, which occur first in the peripheral rod-rich regions and then in the central cone-rich fovea [9]. Heterozygous female carriers are often asymptomatic, and imaging shows RPE mottling due to random X-linked inactivation. Some carriers experience a mild reduction in visual function and show a coarser pattern of degeneration with patches of RPE atrophy. A minority of female carriers develop a male pattern phenotype with the associated significant reduction in retinal sensitivity [10] (Figure 2).

In this manuscript, we briefly summarise choroideremia gene replacement therapy trial results to date and review outcome measures commonly used with a focus on best corrected visual acuity (BCVA). We then cover the role of fundus autofluorescence imaging and its correlations to other structural and functional measures. We discuss the potential use of near-infrared autofluorescence (NIR-AF) imaging in this cohort and explore the melanin-related mechanisms underlying this novel imaging modality.

## 2. Materials and Methods

We conducted a literature search to briefly summarise results from recent clinical trials exploring the safety and efficacy of gene replacement therapy in choroideremia. We report results in terms of their primary outcome measure, which was often defined as a gain in BCVA, and summarise some significant secondary outcome measures. This leads us to then discuss the benefits and limitations of using BCVA as an outcome measure in the cohort of patients with choroideremia and the potential value of using structural outcome measures, such as autofluorescence, as a surrogate for retinal sensitivity.

All images in this manuscript are original. These were collected at the Oxford Eye Hospital, United Kingdom, as part of the screening for a choroideremia gene therapy trial (NCT02407678) approved by the UK Research Ethics Committee (179453). All patients provided informed consent, and the study was conducted in accordance with the Declaration of Helsinki.

## 3. Gene Therapy Trials and Outcome Measures

At present, no approved treatment for choroideremia is available. However, in recent years, there has been notable interest in gene-based therapies for monogenic inherited retinal dystrophies, including choroideremia [11,12,13]. Multiple clinical trials have been completed, and some are currently underway (Table 1). The most studied therapy is gene replacement, where an adeno-associated virus AAV2 vector encoding REP1 is delivered subretinally. Alternative molecular therapies are also being explored both in vitro and in vivo [14]. Early data for gene replacement therapy suggested good safety profiles and encouraging efficacy results [15,16,17], with the first human Phase I/II clinical trial for choroideremia commencing in 2011 in Oxford. AAV2-REP1 was delivered via subretinal injection to 14 male patients. At 24 months, the median BCVA gain in treated eyes was 4.5 letters compared to a 1.5-letter loss in untreated eyes [17]. Four subsequent clinical trials achieved similar results to each other, demonstrating no pronounced gain in BCVA in treated versus untreated eyes at 2 years [18,19,20,21,22]. More recently, the STAR Phase III clinical trial (NCT03496012) did not meet its primary endpoint of improvement of 15 letters in BCVA at 12 months post-sub-retinal AAV2-REP1 injection [23]. This trial would have included mainly participants with fairly advanced choroideremia. The REGENERATE trial (NCT02407678), which included younger participants with earlier stages of degeneration, also found no significant change in BCVA in treated versus control eyes at 24 months [22].

Outcome measures used by clinical trials can broadly be divided into functional and structural ones. Functional measures commonly include BCVA, contrast sensitivity, colour vision, perimetry, and microperimetry, whilst structural measures include spectral-domain optical coherence tomography (OCT) and fundus autofluorescence imaging. The number of gained letters in BCVA testing has been the most commonly used primary outcome measure, together with safety, in choroideremia clinical trials to date. However, due to the nature of the disease, it is recognised that visual acuity is preserved until the late stages of choroideremia, when the degeneration is profound and affects the fovea, with little change in a short period of time. This has been confirmed by longitudinal natural history studies that demonstrated no significant change in visual acuity over a 12-month period [25,26]. In a recent meta-analysis, Shen et al. looked at the long-term natural history of BCVA in 1004 individual eyes of people with choroideremia and found that BCVA followed a 2-phase linear decline, a slower initial phase followed by a rapid decline, with an estimated transition age of approximately 39 years [27]. Therefore, the use of BCVA as an outcome measure may present more challenges in younger patients with less advanced disease when central visual acuity remains fairly stable. In this particular population, structural anatomical measures may be especially valuable. It has also been suggested that low-luminance visual acuity testing may be a useful outcome measure in patients with early to moderate disease when high-contrast acuity remains preserved [28]. Additionally, patients with choroideremia have impaired vision-related quality of life primarily attributable to poor peripheral vision and general vision, with peripheral vision being the worst affected domain in younger individuals, as measured by the National Eye Institute Visual Function Questionnaire (NEI VFQ-25) [26].

Various forms of perimetry testing, which measures sensitivity to visual stimuli at defined points within the visual field, can be used to systematically assess visual field function [29]. More recently, fundus-controlled perimetry, also called microperimetry, has consolidated as a robust functional outcome measure to map visual sensitivity and fixation stability. This is an automated visual field test that combines the technology of the scanning laser ophthalmoscope with an eye-tracking device to allow real-time accurate spatial mapping of the visual sensitivity at specific retinal points [30]. In patients with choroideremia, microperimetry is a valuable outcome measure, given that often visual acuity is preserved until very late [31]. However, a high test-retest variation at the edges of the degeneration has been observed, introducing challenges when monitoring sensitivity changes at these transitional zones and affecting mean sensitivity changes [32]. This may be one of the reasons behind small and inconsistent changes in mean sensitivity measured by microperimetry found by Hagag et al. at 6-month and 12-month intervals in a cohort of patients with choroideremia [26]. A novel method to analyse microperimetry data using volumetric measures instead of mean sensitivity (which suffers from an artificial floor effect in patients with poor vision due to scotomas) was recently developed by Josan et al. [33]. This was able to detect differences in retinal sensitivity, which were not appreciable by mean sensitivity analysis. Hence, it could be of value in future clinical trial analysis. In a recent study, we performed dominance analysis (Shapley regression) to rank visual outcome functional measures in terms of variable importance. These included BCVA using an Early Treatment Diabetic Retinopathy Study (ETDRS) chart, low luminance visual acuity (LLVA), Pelli–Robson contrast sensitivity, Full-threshold stimulus testing (FST), electroretinography (ERG), Cambridge contrast sensitivity function (CSF) and Cambridge colour test (CCT). The strongest association between disease severity (as inferred by the size of the outer retinal island autofluorescence area on BAF) and all functional measure covariates was microperimetry. All other predictors were significantly lower in importance. ETDRS chart BCVA under standard lighting conditions was ranked lowest among the panel of test modalities [34].

It has been observed that the slow functional progression, especially in younger patients with choroideremia, the subjective nature of functional parameters, and visit-to-visit variability may limit the applicability of these functional endpoints to detect short-term gains or losses, which are necessary for the evaluation of novel therapies in clinical trials [26]. Reliable and informative structural measures, which correlate with functional and patient-reported outcome measures, may be of marked value. However, accurate and consistent demarcation remains challenging, using either manual or automated algorithms, and the measurement of these structural endpoints is not always straightforward. Additionally, the need to reconsider outcome measures, with a stronger focus on the preservation of vision by slowing disease progression rather than restoration of lost visual acuity, has been recently raised [11].

## 4. Fundus Autofluorescence

Short-wavelength blue autofluorescence (BAF) is currently widely used and involves the use of a 488 nm laser excitation light to allow in vivo topographic mapping of the lipofuscin fluorophores distribution in the RPE, acting as a surrogate marker of RPE health [35]. Lipofuscin granules are a byproduct of the visual cycle, and their accumulation is considered a cumulative index of oxidative damage and a biomarker of cellular aging [36,37]. Therefore, BAF is increased in the presence of RPE dysfunction and is decreased in areas where the RPE and photoreceptors have been lost [35]. This produces a characteristic appearance in choroideremia with sharply demarcated scalloped edges, which define the area of surviving RPE. The average rate of AF loss, and hence degeneration, has been predicted at 7.7% of the residual retinal area per year and has been observed to follow a logarithmic pattern with age, meaning that the disease progression reduces with age. The degeneration appears to affect the nasal hemiretina faster than the temporal hemiretina, with the fovea being the area of greatest AF preservation [6,38]. A linear relationship has been identified between foveal sensitivity on microperimetry and the distance from the centre of the anatomical fovea to the nearest edge of degeneration on autofluorescence [31]. This is an important prognostic factor that provides an indication of the time before foveal involvement and subsequent sharp decline in visual acuity.

A correspondence has been found between the edge of the BAF island and the start point of a sharp decline in outer nuclear layer (ONL) thickness on OCT, which also qualitatively corresponded to the location where the ellipsoid zone (EZ) terminated. However, the ill definition of the EZ on routine OCT scans at the edges of the degeneration made this a less reliable predictor of the BAF borders [39]. Using custom OCT software, Hariri et al. found a high correlation between areas of preserved EZ and BAF, with the EZ area being significantly larger than the BAF area [40]. Interestingly, three distinct RPE patterns can be observed on BAF imaging in some patients with choroideremia, especially in the earlier disease stages: (i) smooth areas with homogenous normal appearance, (ii) mottled areas with a granular mixed hyper and hypo-autofluorescent appearance, and (iii) atrophic areas with complete loss of autofluorescence (Figure 1). These have been found to correlate with the health of the EZ on OCT. Smooth regions mostly contain intact EZ and RPE, whilst mottled regions are associated with largely disrupted EZ [41]. As well as displaying preserved anatomical features, smooth zones have also been associated with near-normal retinal sensitivity as determined by microperimetry. Meanwhile, whilst areas with a mottled appearance on BAF still retain retinal sensitivity, this has been found to be measurably impaired compared to age-matched controls [42]. These ovoid smooth zones spatially correspond to the areas of highest cone photoreceptor density [43], where the cone mosaic is intact with either normal or reduced cone density [5]. The preservation of the retinal architecture in these areas supports the hypothesis that cone photoreceptors are less affected by REP1 deficiency. Hence, in some patients with choroideremia, two BAF patterns can be observed: (1) an outer ring with a mottled appearance where RPE cells are surviving but dysfunctional, and (2) an inner smooth area with healthy RPE cells, which are able to support overlying photoreceptors.

An alternative AF imaging approach is NIR-AF. This uses a 787 nm near-infrared light to visualise the distribution of melanin, a fluorophore mainly originating from the RPE and, to a varying degree, from the underlying choroid [44]. Melanosomes in the RPE and the choroid have indeed been confirmed as the dominant source of NIR-AF in the posterior region of the eye in an ex vivo study [45]. NIR-AF is a less commonly used imaging modality that has recently undergone more evaluation and has shown potential for use in disease assessment and progression monitoring in patients with retinitis pigmentosa [46]. Birtel et al. first systematically investigated NIR-AF characteristics in a cohort of 43 patients with choroideremia, exploring its relation to structural and functional parameters [47]. They identified three phenotypic patterns of NIR-AF: (i) areas of normal/homogenous preservation, (ii) areas with granular/mottled disruption, and (iii) areas with complete loss of RPE-related NIR-AF, where there is unmasking of the underlying choroid and some background signal arising from the choroidal stroma. Interestingly, on qualitative comparison in earlier disease stages, they found that the pattern of homogenous preservation of NIR-AF visually corresponded to areas of normal smooth appearance on BAF imaging rather than corresponding to the whole area of the residual BAF island. On analysis of quantitative structural measures, it was observed that the width of the EZ was similar to the horizontal diameter of the residual NIR-AF island for participants who displayed all three NIR-AF patterns. Participants who only had small areas of preserved homogenous NIR-AF without any area of granular pattern had a wider EZ compared to the diameter of the area with homogenous NIR-AF. This may be explained by the fact that NIR-AF correlates to the area of smooth BAF rather than the whole BAF island, and the EZ (intact or disrupted) has been shown to extend to the edges of the BAF island, irrespective of its smooth or mottled appearance [39]. The correspondence described between different structural parameters, including homogenous NIR-AF, smooth BAF, and intact EZ, would support the hypothesis that these regions displaying both smooth BAF and homogenous NIR-AF represent areas of healthy retina and RPE integrity.

Birtel et al. also characterised the correlation between NIR-AF appearance and visual function, measured by BCVA and the point with the highest sensitivity at or around the fixation point in microperimetry [47]. Whilst they found no statistical difference in BCVA between those exhibiting different NIR-AF patterns, there was a correlation between the maximal foveal sensitivity on microperimetry and the pattern of residual NIR-AF. Patients with preserved normal homogenous NIR-AF signal had higher maximal foveal sensitivity compared to those who only had disrupted or absent NIR-AF. They observed that the residual island on BAF better represented the area of preserved retinal function on microperimetry compared to NIR-AF. This implies that areas with no NIR-AF but that maintain a mottled BAF signal are still functioning. However, how the retinal function compares between areas that maintain both NIR-AF and BAF properties and areas that have lost their NIR-AF but are still visible on BAF remains unclear at present. Considering the significant phenotypical variation between affected individuals, evaluation of autofluorescence patterns in patients who still have significantly preserved visual function, including children with choroideremia or choroideremia female carriers, in relation to the presence or absence of a smooth zone on BAF and homogenous NIR-AF, or whether the total island size on BAF irrespective of pattern is more indicative of overall visual function, would be worthwhile.

## 5. The Role of Melanin

The RPE is constituted by a monolayer of highly pigmented cells located posterior to the photoreceptors of the neurosensory retina and line the inner aspect of Bruch’s membrane. Its metabolic and transport functions are critical for neuronal homeostasis, and its dysfunction has been implicated in various retinal diseases [48]. RPE cells are nonregenerative and must, therefore, last to preserve vision [49]. The human RPE contains predominantly three types of pigment granules: melanosomes, lipofuscin, and melanolipofuscin. RPE melanosomes are synthesized in utero during a brief window of embryogenesis and achieve maturation in early postnatal life. Virtually no melanin renewal is thought to occur, evidenced by the lack of tyrosinase activity in fully matured melanosomes, an enzyme that is essential for melanin biosynthesis [50,51]. On the contrary, lipofuscin granules accumulate as a result of the ageing process, and melanolipofuscin granules are characteristic of aged RPE. Melanin is an insoluble, high-molecular-weight polymer derived from the enzymatic oxidation of tyrosine and dihydroxyphenylalanine, which is synthesized and stored within melanosomes in pigmented cells [52]. Melanin in the RPE is believed to have a protective role against light damage and oxidative stress [49,53,54,55]. A consistent reduction in melanin content in RPE cells with age has been observed, which may be attributable to the significant age-related photochemical modification, light-induced irradiation, and lysosomal degradation that affect melanosomes [55]. These processes change the melanosomes’ spectral characteristics and photoreactivity, reducing their antioxidation and photoprotective capacity [56,57,58]. The density of RPE melanosomes is also thought to reduce as lipofuscin accumulates as a result of age or disease, where the melanosomes fuse with lipofuscin granules to form melanolipofuscin [52,59].

**Figure 3:**
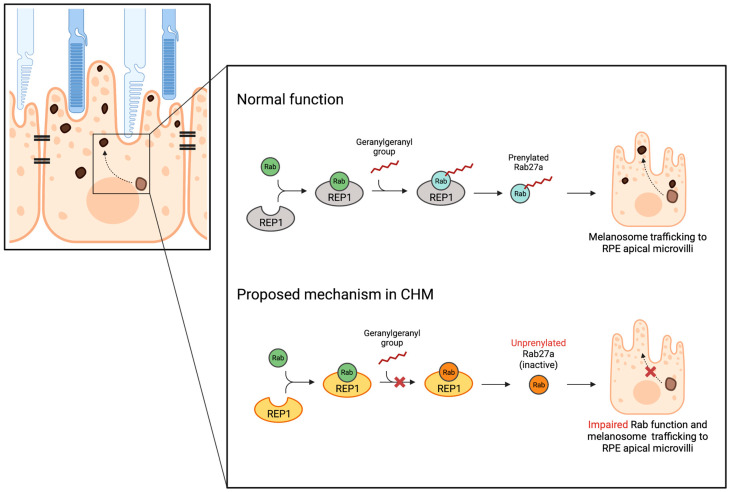
A schematic representation of the Rab prenylation pathway and effect on RPE melanin. REP1 (Rab Escort Protein 1) binds the newly synthesized Rabs (Ras-related proteins) and mediates the addition of a geranylgeranyl diphosphate group to the Rab C-terminus, resulting in prenylation. Rab27a is thought to have a role in melanosome transport, and its correct functioning is required for the light-dependent movement of melanosomes into the apical processes within RPE cells.

The REP1 protein, which is defective in choroideremia, is one of two REP isoforms required for the attachment of geranylgeranyl groups to Rab proteins, without which these remain inactive and unprenylated [60,61]. Rab proteins are GTPases that regulate intracellular vesicular trafficking, especially docking, and fusion of vesicles that cause the release of contents from the transport vesicle into the receptor organelle [62,63]. Many Rab proteins have been observed to be normally prenylated in choroideremia. However, Rab27 has been found to preferentially depend on REP1 for prenylation and has, therefore, been suggested to be potentially implicated in the retinal degeneration seen in this disease [61]. The fact that Rab27 was found to selectively accumulate in the cytosol of choroideremia lymphoblasts, whilst it normally is membrane-bound, suggests compromised function in this disease. Additionally, it was found to be expressed in the RPE and choriocapillaris, the layers that characteristically degenerate in choroideremia [61]. Rab27a is thought to have a role in melanosome transport [64] and is required for the light-dependent movement of melanosomes into the apical processes within RPE cells [65]. Therefore, these observations may point toward a defect in the RPE melanin metabolism in choroideremia (proposed mechanism illustrated in Figure 3).

## 6. Distinct Retinal Patterns on Autofluorescence Imaging

The combination of possible defects in melanin transport consequent to REP1 deficiency and the likely additional insults to the melanosomes occurring as a result of RPE dysfunction and degeneration in choroideremia may result in accelerated loss of melanin in the RPE. As the RPE cells lose their melanosomes, they are likely to lose the protective effects provided by melanin, hence being more susceptible to light-induced oxygen-reactive species and consequently suffering higher levels of damage. Therefore, the loss of melanin might precede the death of the RPE cells, and it may especially precede the accumulation of lipofuscin and melanolipofuscin granules within them. This may help us interpret the data we obtain from different autofluorescence imaging modalities.

**Figure 4:**
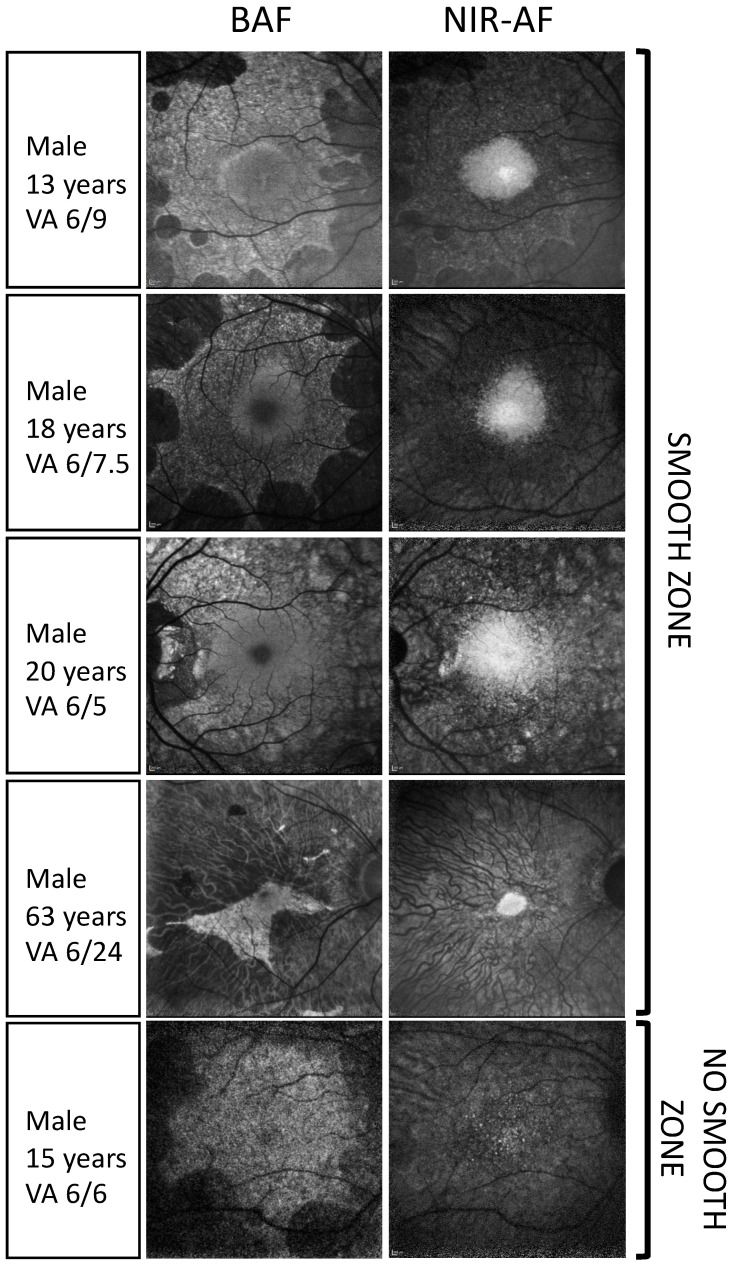
Examples of corresponding 30° short wavelength blue autofluorescence (BAF) images and 30° near-infrared autofluorescence (NIR-AF) images for four patients with choroideremia ranging from children to older individuals, with varying phenotypes and visual acuities. These demonstrate qualitative visual correspondence between areas with a smooth appearance on BAF and areas with preserved homogenous NIR-AF (top four panels). The bottom panel is an example of a young patient with excellent visual acuity but without smooth zones on BAF and NIR-AF despite a large residual island.

As previously described [39,41], we have observed that a proportion of patients with choroideremia have ovoid areas of smooth BAF signal, commonly located around the fovea and surrounded to a varying degree by areas of mottled BAF signal. As pointed out by Birtel et al. [47], there appears to be a qualitative correspondence between zones that appear smooth on BAF and areas that have a homogenous NIR-AF signal (shown in Figure 4). We can hypothesise that these are areas where the melanin is still preserved in such a way that it maintains its protective effect and NIR-AF signal and where there is not yet a pathological accumulation of lipofuscin granules or RPE loss causing a mottled mixed hyper- and hypo-fluorescent pattern on BAF imaging. In essence, a smooth appearance on BAF and a homogenous pattern on NIR-AF may indicate those areas of near normal retina and intact RPE [42] (Figure 4).

**Figure 5:**
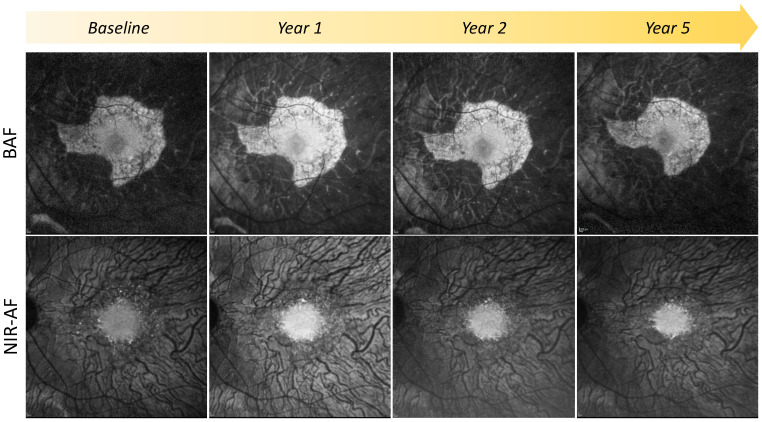
Longitudinal changes in 30° BAF and 30° NIR-AF imaging over a five-year follow-up period demonstrate slow progression over time. Correspondence of preserved NIR-AF and BAF smooth pattern is maintained throughout follow-up, with a qualitative reduction in the overall retinal island on BAF, as well as the smooth region on BAF, and are of homogenous NIR-AF.

In contrast, there are areas towards the edges of the retinal degeneration that have lost the NIR-AF signal but retain the BAF signal, despite this being granular mottled, and still have a measurable ellipsoid zone, although this may be disrupted [41]. Therefore, the loss of melanin-related NIR-AF appears to precede the loss of RPE (as measured by BAF) and photoreceptors (indicated by the presence of an ellipsoid zone on OCT) [47]. Figure 5 demonstrates an example of how BAF and NIR-AF imaging change in a patient over a 5-year follow-up. Similar findings have been observed in patients with retinitis pigmentosa [46] and Stargardt disease [66], suggesting that possibly the loss of NIR-AF precedes the formation of lipofuscin fluorophores visible on BAF. The loss of NIR-AF may signify a specific early stage of degeneration where the altered RPE has lost its melanin content and produces lipofuscin at accelerated levels. There might also be an interactive effect between the melanosomes and the lipofuscin fluorophores, where the increased fractional volume of lipofuscin might alter the NIR-AF properties of the interspersed melanin [46]. Functionally, in patients with choroideremia, these areas with no NIR-AF but mottled BAF retain retinal sensitivity on microperimetry testing [47]. In contrast, outside the border of the surviving RPE island, a sharp decline in visual sensitivity has been observed [67], also mirrored by an abrupt loss of the cone mosaic [5]. To which degree visual function remains preserved in areas with absent NIR-AF but mottled BAF and how this compares to areas that retain both intact NIR-AF and BAF remains unknown.

## 7. Conclusions

These emerging patterns in autofluorescence imaging in choroideremia are interesting observations that may provide valuable insight into the natural history of the disease and aid in the clinical evaluation of patients. A multimodal imaging approach to include NIR-AF as well as routine BAF for the evaluation of patients with choroideremia may improve disease assessment and monitoring.

Additionally, with the advent of therapeutic avenues including but not limited to gene replacement therapy, a better characterisation of the area of the preserved retina, which maintains a healthy RPE, may be of particular value. Gene therapy in choroideremia requires areas of relatively preserved retinal architecture, such that AAV2-REP1 can transduce in surviving RPE and photoreceptor cells, resulting in the production of functioning REP1 and consequent prenylation activity [68]. For late-stage degeneration, vision restoration that includes gene-agnostic approaches would be more suitable [69,70]. Current trials have targeted areas of surviving retina as defined by the central islands identified on BAF. The significance of the smooth retinal pattern seen on BAF and its correspondence to areas with homogenous NIR-AF remains unclear at present. These areas may represent the zone where RPE and retinal structure are still largely unaffected, whilst areas with mottled BAF and absent NIR-AF may be actively degenerating and possibly past the point of rescue by gene therapy. Retinal patterns seen on autofluorescence and the role of NIR-AF might be valuable in the assessment of novel therapies.

## Figures and Tables

**Table 1:** Summary of gene replacement therapy clinical trials in choroideremia.

Study Details	Centre	Start	Vector	Outcomes
NCT01461213 Phase I/II	University of Oxford, UK	2011 Completed	r.AAV2-REP1	At 2 years: median BCVA gain +4.5 letters (statistically significant) (*n* = 14) [15,16,17].
NCT02341807 Phase I/II	Philadelphia, USA Spark Therapeutics	2015 Completed	AAV2-hCHM	At 2 years: BCVA within 15 letters of baseline in 13 of 15 participants. No significant differences between the control and intervention eye in sensitivity by perimetry or RPE preservation by OCT or BAF (*n* = 15) [21].
NCT02077361 Phase I/II	University of Alberta, Canada	2015 Completed	r.AAV2-REP1	At 2 years: no significant gain in BCVA compared to baseline in five of six treated eyes (one subject had an 8-ETDRS-letter loss and one subject had a >15 letter gain). Similar rate of decline in preserved RPE area by BAF between treated and untreated eyes (*n* = 6) [18]. At 4.5 years: non-significant mean BCVA change from baseline −0.2 vs. −3.2 letter in treated vs. untreated (*n* = 5) [24].
NCT02553135 Phase II	University of Miami, USA	2015 Completed	r.AAV2-REP1	At 2 years: BCVA +/− 2 letters in four of six treated eyes (one gained +10 letters and another +5 letters) (*n* = 6) [19].
NCT02671539 THOR TRIAL Phase II	University of Tubingen, Germany STZ eye trial	2016 Completed	r.AAV2-REP1	At 2 years: non-significant mean BCVA gain +3.7 letters (*n* = 6) [20].
NCT02407678 REGENERATE TRIAL Phase II	University of Oxford and Moorfields Eye Hospital, UK	2016 Completed	r.AAV2-REP1	At 2 years: no statistically different comparative change from baseline in BCVA between treated eyes (−2.63 letters, SE 2.76) and control eyes (+2.67 letters, SE 0.768) (*n* = 30) [22].
NCT03496012 STAR TRIAL Phase III	NightstaRx, a Biogen Company International multi-centre	2017 Completed	r.AAV2-REP1 (BIIB111)	At 12 months: failed to meet primary endpoint (proportion of participants with >/= 15 letter gain BCVA) or key secondary endpoints (*n* = 170) [23].
NCT03507686 GEMINI TRIAL Phase II	NightstaRx, a Biogen Company International multi-centre	2017 Completed	r.AAV2-REP1 (BIIB111)	No report to date
NCT04483440 Phase I	4D Molecular Therapeutics, USA	2020 Ongoing	4D-110	No report to date

## Data Availability

The original contributions presented in this study are included in the article. Further inquiries can be directed at the corresponding author.
